# Prospective, Single-Arm Pivotal Study for the Treatment of Subjects With Severe Symptomatic Calcific Aortic Valve Stenosis Using the Valvosoft Noninvasive Ultrasound Therapy

**DOI:** 10.1161/CIRCULATIONAHA.125.077582

**Published:** 2026-08-11

**Authors:** Flavien Vincent, Hélène Eltchaninoff, Bernard Iung, Marleen Van Wely, Etienne Puymirat, Menno van Gameren, Laurent Faroux, Giovanni Amoroso, Won-Keun Kim, Eric Van Belle, Gaspard Suc, Osama Soliman, Bernard Cholley, Danijela Trifunović-Zamaklar, Laurent Lepage, Dimitry Schewel, Alexander Ijsselmuiden, Mathieu Pernot, Michael Tanter, Christian Spaulding, Emmanuel Messas

**Affiliations:** 1 Cardiology, University Hospital Lille, France (F.V., E.V.B.).; 2 Université Rouen Normandie, Inserm U1096, CHU Rouen, Department of Cardiology, Institut Alain Cribier, France (H.E.).; 3 Department of Cardiology, CHU Rouen, Rouen, France (H.E.).; 4 Cardiology, Hôpital Bichat, Paris, France (B.I., G.S.).; 5 INSERM LVTS1148, Université Paris Cité, France (B.I., G.S.).; 6 Cardiology, Radboud University Medical Centre Nijmegen, the Netherlands (M.V.W.).; 7 Cardiovascular Department, Hôpital Européen Georges-Pompidou, Université Paris-Cité, France (E.P., C.S., E.M.).; 8 INSERM Centre d’Investigation 1418, Paris, France (E.P., C.S.).; 9 Cardiology, Amphia Hospital, Breda, the Netherlands (M.v.G.).; 10 Cardiovascular Department, Robert Debré Hospital University Hospital Reims, France (L.F.).; 11 Cardiovascular Department, Claude Cabrol Hospital, University Hospital Reims, France (L.F.).; 12 Cardiology, Onze Lieve Vrouw Gasthuis Amsterdam, the Netherlands (G.A.).; 13 Cardiology, Kerckhoff Klinik, Bad Nauheim, Germany (W.-K.K.).; 14 CORRIB Research Centre for Advanced Imaging and Core Laboratory, University of Galway, Ireland (O.S.).; 15 Cardiovascular Research Institute Dublin (CVRI Dublin), Mater Private Network and RCSI University of Medicine and Health Sciences, Ireland (O.S.).; 16 Department of Anesthesiology, Intensive Care and Perioperative Medicine, Hôpital Européen Georges-Pompidou, AP-HP, Paris, France (B.C.).; 17 Faculty of Medicine, University of Belgrade, Serbia (D.T.-Z.).; 18 Cardiology, Clinique Pasteur, Toulouse, France (L.L.).; 19 Cardiology, Marienkrankenhaus, Hamburg, Germany (D.S.).; 20 Department of Interventional Cardiology, Maastricht University Medical Center/Zuyderland Hospital, the Netherlands (A.I.).; 21 Physics for Medicine Paris, Inserm/ESPCI Paris-PSL/CNRS, France (M.P., M.T.).; 22 Inserm UMR U970 Paris France by Université Paris-Cité, INSERM, France (E.M.).

Calcific aortic stenosis is a progressive disease with limited treatment options for patients unsuitable for surgical or transcatheter aortic valve replacement because of frailty, comorbidities, or limited life expectancy. Noninvasive ultrasound therapy (NIUT) represents a new strategy that uses externally delivered focused ultrasound beams to soften calcified valve leaflets and to restore mobility. Valvosoft (Cardiawave, Levallois-Perret, France), CE marked in 2025, is the first noninvasive system specifically developed for this purpose (Figure [A–C]).^1–3^ We report the first 12-month outcomes of the Pivotal study in patients with severe calcified aortic stenosis deemed ineligible for transcatheter aortic valve replacement.

**Figure.**
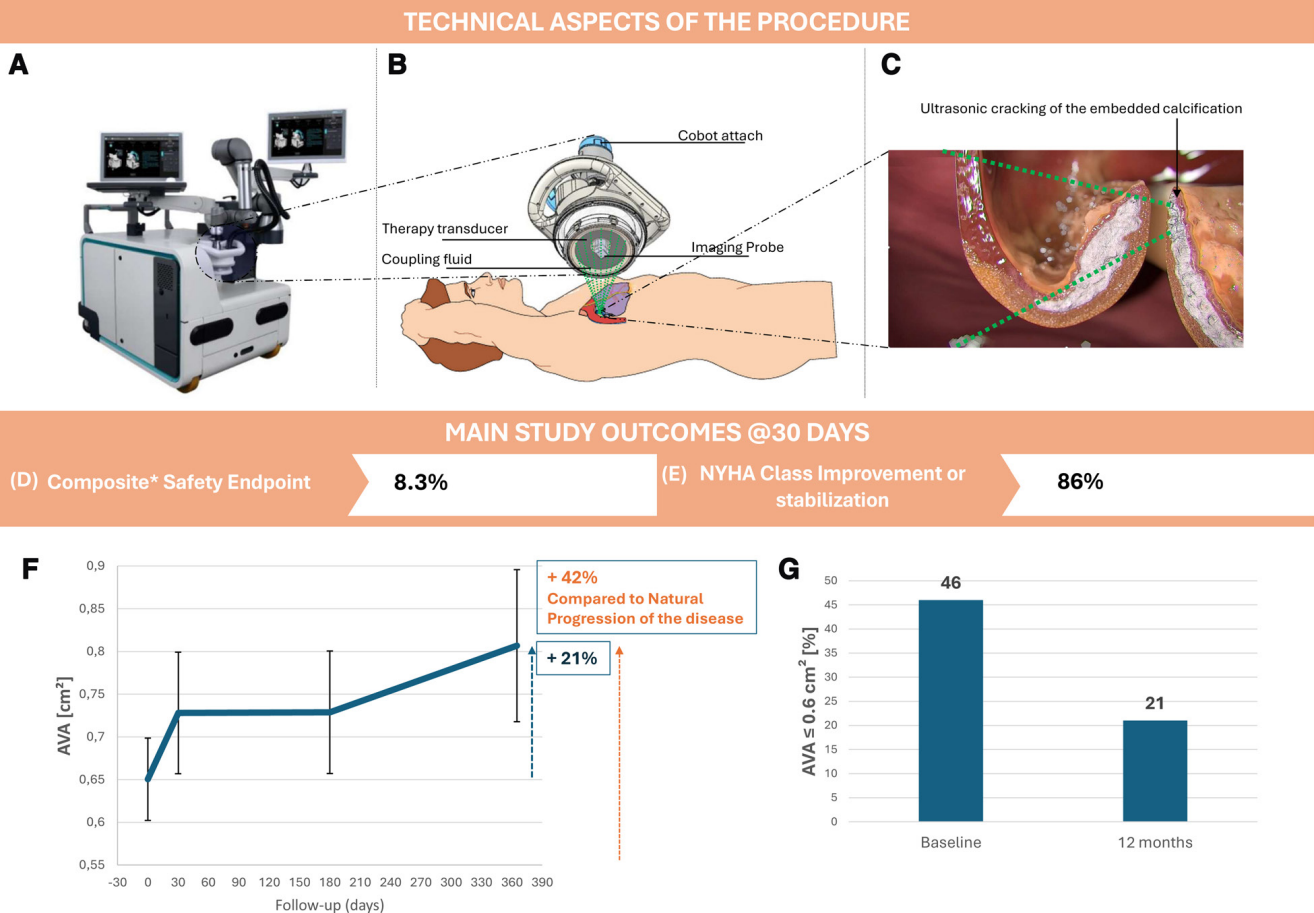
**Technical aspects and clinical outcomes of the noninvasive ultrasound therapy procedure**. The Valvosoft device is a novel noninvasive, real-time image-guided device that uses focused ultrasound to induce cavitation. This mechanical softening increases leaflet mobility without damaging surrounding tissue. The second-generation Valvosoft system incorporates enhanced biplane imaging to improve targeting precision and procedural ergonomics. **A**, System components include the therapeutical ultrasound generator and amplifier, the imaging system, and an applicator. **B**, An ultrasound imaging probe positioned centrally within the applicator enables visualization of the aortic valve leaflets and continuous real-time therapy monitoring. The applicator is placed on the patient’s chest. When cavitation bubbles collapse, they generate shockwaves capable of fracturing calcifications within the valve leaflets. Individual beams passing through tissue have no therapeutic effect. However, at the focal point, the convergence of multiple beams produces the desired physiological effects (**C**). The primary safety end point (**D**), a 30-day composite* of all-cause mortality, myocardial infarction, coronary revascularization, stroke, major and life-threatening bleeding, and hospitalization for heart failure, was observed in 5 patients: 3 deaths (sudden death, sepsis, and progressive decline related to underlying preexisting conditions, all classified as cardiovascular deaths by the Clinical Events Committee), 1 myocardial infarction (transient, mild increase in troponin and electrocardiographic changes; coronary angiography revealed no acute coronary artery occlusion, and cardiac magnetic resonance imaging showed no ischemia or infarction; the Clinical Events Committee adjudicated the case as myocardial infarction), and 1 life-threatening bleeding after a fall. **E**, The primary performance end point was the rate of New York Heart Association (NYHA) class improvement at 30 days (43%). **F**, Paired data (n=24). Improvement of 21% compared with baseline and 42% compared with the natural progression of the disease. The low number of echocardiographic assessments at follow-up reflects the multimorbid and frail patient population, many of whom either died or were unable or unwilling to return for follow-up imaging. In addition, imaging quality was inadequate in some cases. **G**, Percent of patients with aortic valve area (AVA) ≤0.6 cm^2^.

The trial was approved by the responsible ethics committee, and all patients signed informed consent. Methods are provided at ClinicalTrials.gov. Data will be available on reasonable requests.

This prospective single-arm study enrolled 60 patients across 11 centers from June 2022 to July 2023. Median age was 88 years (interquartile range [IQR], 82–91 years); 65% (39/60) of patients were female. Median Society of Thoracic Surgeons and EuroSCORE II scores were 5.3% (IQR, 3.5%–9.6%) and 5.8% (IQR, 2.7%–8.7%), respectively. Atrial fibrillation was present in 37% (22/60), heart failure in 58% (35/60), chronic kidney disease in 38% (23/60), prior cerebrovascular accidents in 27% (16/60), carcinoma in 25% (15/60), and chronic obstructive pulmonary disease in 15% (9/60); 46.7% (28/60) had a Clinical Frailty Scale score ≥6. Only 34% (20/59) had high-gradient aortic stenosis; 66% (39/59) had low-gradient disease; and 7% (4/59) had bicuspid valves.

Each NIUT session was up to 70 minutes of energy divided in cycles of 10 minutes maximum, allowing target zone repositioning between cycles. No specific medication regimen was required. The procedure was performed in various settings, including the echocardiography laboratory and ambulatory operating room. It was feasible in all patients and was performed under mild conscious sedation (n=46) or analgesia (n=13) for patient comfort; 1 patient received general anesthesia. The median procedure time was 70 minutes (IQR, 70–70 minutes). Procedural tolerance was excellent: Mild pain occurred in 7 patients, and mild, self-limiting arrhythmias (not requiring intervention) occurred in 3 patients.

All patients were discharged alive, and the modified Rankin Scale score remained stable through 30 days (median, 2.0 [IQR, 0–3] at baseline, discharge, and 30 days; n=54 paired data).

The primary safety end point, a composite of all-cause mortality, myocardial infarction, coronary revascularization, stroke, major and life-threatening bleeding, and heart failure hospitalization at 30 days, met the prespecified threshold, with the upper CI bound below the 25% event rate considered acceptable for this frail population (8.3% [95% CI, 2.8%–18.4%]). All events except 1 (a transient troponin elevation without evidence of myocardial ischemia) were attributable to patients’ comorbidities (Figure [D]). The transient troponin increase may have been related to cavitation; however, focal spot steering and real-time monitoring were implemented to allow therapeutic precision and to protect myocardial tissue.

Despite multiple comorbidities, an improvement or stabilization in New York Heart Association class at 30 days was obtained in 86% of patients (primary performance end point improvement in New York Heart Association class in 43%; Figure [E]). Furthermore, the median Kansas City Cardiomyopathy Questionnaire score improved by 7 points, a change considered clinically meaningful.

Instead of the expected progressive reduction in aortic valve area (AVA), reflecting the natural progression of the disease, we observed an increase in AVA over time, suggesting a form of reverse valvular remodeling. Similarly, research is exploring pharmacological strategies to target calcific aortic valve stenosis progression (ClinicalTrials.gov, NCT05646381).^4^ In our study, AVA improved from 0.67±0.22 cm^2^ at baseline to 0.81±0.24cm^2^ at 12 months (*P*<0.001 [n=24], Wilcoxon signed-rank test; Figure [F]); mean gradient improved from 37.0±12.6 to 34.3±11.6 mm Hg (*P*=0.096 [N=29], acknowledging that 66% of patients had baseline low gradient stenosis), and stroke volume index improved from 34.2±9.9 to 40.9±13.9 mL/m^2^ (*P*<0.001; n=21).

AVA continued to increase beyond 30 days, suggesting NIUT produces an immediate mechanical effect followed by delayed biological effects, consistent with preclinical findings.^3^ These significant improvements led to a reduced proportion of patient with AVA ≤0.6 cm^2^, a threshold strongly associated with mortality and adverse events.^5^

To account for the misses in paired 12-month data, an analysis up to the last follow-up was conducted, showing a similar pattern. At a median follow-up of 361 days (IQR, 89–376 days), AVA improved from 0.66±0.18 to 0.77±0.24 cm^2^ (*P*<0.001 [n=49]), mean gradient improved from 36.3±13.1 to 33.4±12.8 mm Hg (*P*=0.050 [n=52]), and stroke volume index improved from 33.4±10.6 to 37.9±13.4 mL/m^2^ (*P*<0.001 [n=49]).

Limitations include the single-arm design and the small number of echocardiographic assessments due to the frail and comorbid population (median age, 88 years). At the 12-month follow-up, 31 echocardiograms were available; 21 patients had died, 1 withdrew consent, and 7 missed follow-up visits.

In conclusion, NIUT is a safe procedure with an absence of cerebrovascular events that can be performed outside of the catheterization laboratory. Beyond treating patients unsuitable for transcatheter aortic valve replacement, NIUT may serve as bridge or adjunct to transcatheter aortic valve replacement to improve prosthesis expansion and to reduce complications and as treatment for moderate aortic stenosis to slow disease progression. These benefits warrant confirmation in ongoing clinical studies.

## Article Information

### Acknowledgments

The authors thank Beatrix Doerr, medical writer, for her help in preparing this manuscript. This work is part of the program RHU STOP-AS “Investissements d’avenir” with the reference ANR-16-RHUS-0003, managed by the National Research Agency.

### Disclosures

Dr Kim reports honoraria from Abbott, Boston Scientific, Edwards Lifesciences, Jenavalve, Meril Life Sciences, Anteris, P & F, and HID Imaging and participates in the Data Safety Monitory Board/Advisory Board of the P&F VIVA study. Dr Soliman reports institutional research grants from industry (no personal remuneration), national and EU commercialization grants (EIC Transition, DTIF), and service as chair of the PRECISE Imaging Core Lab and Chair of Data Safety Monitory Board/Clinical Events Committee in industry-sponsored trials. Dr Trifunović-Zamaklar reports grants for the Researcher project SINERGY-ACUTE (7558), within the PRISMA program Science Fund of the Republic of Serbia (2024-2027), for ECHOS3 from AstraZeneca (payments to the national echocardiographic society); consulting fees from CERC; and lecturer fees from Boehringer Ingelheim, AstraZeneca, Pfizer, Krka, Novartis, and MSD. Dr Trifunović-Zamaklar participates in Data Safety Monitory Board/Advisory Board meetings from Boehringer Ingelheim Serbia and is the President of the Cardiology section of the Serbian Medical Society and a past President of the Echocardiographic Society of Serbia. Dr Lepage reports grants from General Electric Healthcare. Drs Pernot, Tanter, and Messas are cofounders and shareholders of Cardiawave. Dr Spaulding reports no conflicts for this topic and as other conflicts as follows: grants and research support from CERC and French Ministry of Health; honoraria from Sonivie, Techwald, Novartis, Sanofi, Medtronic, Valcare, and Boston Scientific; and stock options in Sonivie and Techwald. Dr Messas reports research grants from Novartis paid to his institution. The other authors report no conflicts.
